# Changes in Cross-Sectional Area and Transverse Diameter of the Heart on Inspiratory and Expiratory Chest CT: Correlation with Changes in Lung Size and Influence on Cardiothoracic Ratio Measurement

**DOI:** 10.1371/journal.pone.0131902

**Published:** 2015-07-07

**Authors:** Hayato Tomita, Tsuneo Yamashiro, Shin Matsuoka, Shoichiro Matsushita, Yasuyuki Kurihara, Yasuo Nakajima

**Affiliations:** 1 Department of Radiology, St. Marianna University School of Medicine, Kawasaki, Kanagawa, Japan; 2 Department of Radiology, Graduate School of Medical Science, University of the Ryukyus, Nishihara, Okinawa, Japan; University of Athens Medical School, GREECE

## Abstract

**Objective:**

The aim of this study was to investigate physiological changes in cardiac area and diameters between inspiratory and expiratory chest computed tomography (CT), and to assess their correlation with lung size change and influence on cardiothoracic ratio (CTR) measurements.

**Materials and Methods:**

The institutional review board of our institution approved this study, and informed consent was waived. Forty-three subjects underwent inspiratory and expiratory chest CT as part of routine clinical care. On both inspiratory and expiratory scans, lung volumes and maximum lung diameters (transverse and vertical directions) were measured. The maximum cardiac cross-sectional area (CSA) and the maximum transverse cardiac diameter were measured on both scans, and the CT-based CTR was calculated. Changes in the lung and cardiac measurements were expressed as the expiratory/inspiratory (E/I) ratios. Comparisons between inspiratory and expiratory measurements were made by the Wilcoxon signed-rank test. Correlations between the E/I ratios of lung and heart measurements were evaluated by Spearman’s rank correlation analysis.

**Results:**

Cardiac CSA and transverse cardiac diameter was significantly larger on expiratory than on inspiratory CT (*p *< 0.0001). Significant negative correlations were found between the E/I ratios of these cardiac measurements and the E/I ratios of lung volume and vertical lung diameter (*p *< 0.01). CT-based CTR was significantly larger on expiration than on inspiration (*p *< 0.0001).

**Conclusions:**

Heart size on chest CT depends on the phase of ventilation, and is correlated with changes in lung volume and craniocaudal lung diameter. The CTR is also significantly influenced by ventilation.

## Introduction

The cardio-thoracic ratio (CTR), usually calculated using chest radiographs, is a widely used radiographic parameter as an index of heart size. CTR greater than 50% is generally judged as cardiomegaly [1]; however, it has been reported that the clinical value of CTR measurement is very limited for screening heart failure or true cardiomegaly [2], since CTR is based on transverse diameters only, and does not assess the cardiac diameter in the anterior-posterior direction [3,4]. However, CTR measurement is still recommended in the guideline for management of the patients with congenital heart diseases [5], since it is known that longitudinal changes in CTR precisely reflect exacerbation or improvement of cardiomegaly [6–10]. With the development of computed tomography (CT), CTR measurement using chest CT has been attempted in some researches, showing that very high correlations and minimum differences between CTR on chest radiographs and that on chest CT [11].

It is easy to understand how the cardiothoracic ratio (CTR) would be influenced by thoracic movements or ventilation, since the diameters of the thorax must change from inspiration to expiration with expansion and contraction of the chest wall. However, there is no previous research that demonstrates the impact of lung size or the phase of respiration on the CTR. Interestingly, a large change in the CTR between inspiration and expiration has been described in a textbook of pediatric radiology; and this phenomenon was suggested as a confoundable finding in the evaluation of congenital heart diseases, since infants or children cannot hold their breath during CT scanning [12]. This textbook used the case of a crying infant to show that the thoracic shape and size greatly change between inspiration and expiration, which also affects the heart shape and transverse cardiac diameter [12]. These changes should result in larger CTR at full expiration than that at full inspiration, and a large difference in the CTR based on CT compared with CTR based on chest radiography. However, to the best of our knowledge, no previous research exists that clarify the association between respiratory phase and CTR measurements in adults.

Recently, inspiratory and expiratory chest CT has frequently been performed to assess various obstructive diseases. It has already been demonstrated that quantitatively measured lung volume on inspiratory CT strongly correlates with total lung capacity (TLC) measured by pulmonary function testing, and lung volume on expiratory CT correlates with residual lung volume [13–15]. Considering that CT-CTR and lung volume are measurable on inspiratory and expiratory chest CT, it is reasonable to use these CT scans to investigate the influence of ventilation or chest wall movement on heart size and CTR.

In this study, we hypothesized that cardiac area and diameters would change as a physiological phenomenon between inspiratory and expiratory CT scans, and that the CTR would also differ between inspiration and expiration. In addition, we hypothesized that these changes in cardiac areas and diameters would be correlated with changes in lung size. Thus, the aims of this study are, (i) to clarify how cardiac measurements and the CTR change from inspiration to expiration, and (ii) to investigate the correlation between changes in cardiac parameters and changes in lung size using inspiratory and expiratory CT scans.

## Materials and Methods

This retrospective study was approved by Institutional Review Board of St. Marianna University School of Medicine, which waived the need for informed consent from patients. All medical records were anonymized.

### Subjects

A total of 50 subjects were initially identified by a radiologist (H.T., with a three-year experience in thoracic radiology). From April to May 2013, these subjects underwent inspiratory and expiratory chest CT during a single visit to our institution. The subjects had known or probable chest diseases, such as pulmonary emphysema, chronic obstructive pulmonary disease (COPD), bronchial asthma, bronchial tuberculosis or bronchial tumors. In our institution, additional expiratory chest CT is frequently performed upon request by physicians, in order to assess conditions that may show proximal airway narrowing or air-trapping in expiration.

After reviewing the CT data and medical records for these patients, seven patients were excluded from the study due to the following reasons: pericardial effusion or severe cardiomegaly (n = 3), large atelectasis (n = 1), insufficient expiration during expiratory scanning (n = 1), motion artifact (n = 1), and use of contrast medium (n = 1). Thus, a total of 43 subjects were ultimately included in this study. They were 33 males and 10 females (mean age, 68 years; age range, 17–89 years). All patients also underwent plain chest radiography around the time of CT scanning (within two months of chest CT).

### Chest radiography

Standard postero-anterior chest radiography was performed at full inspiration by a Canon CXDI-40EG digital radiography system (Medical Systems Division of Canon U.S.A., Irvine, CA). Chest radiography settings were as follows: distance between the patient and image intensifier, 200 cm; tube voltage, 130kVp; tube current, 250mA.

### CT scanning

All patients underwent plain chest CT by identical 64-row MDCT scanners (Aquilion 64, Toshiba Medical Systems, Otawara, Tochigi, Japan) at full inspiration and end-expiration with a breath hold. CT scanning settings for inspiratory scans were as follows: collimation, 64 × 0.5 mm; tube voltage, 120 kVp; tube current, 200 mA; gantry rotation time, 0.5 sec; beam pitch, 0.828 (helical pitch, 53). Scanning settings for expiratory scans were similar to inspiratory scans, except for a reduced tube current setting (80 mA). Inspiratory and expiratory scan data were converted to CT images using a soft tissue kernel (FC04) with a slice thickness of 7 mm without overlapping of section intervals. The imaging field of view (FOV) was 320 × 320 mm, and the pixel size was 0.625 × 0.625 mm.

### Image analysis

#### (i) Measurement of the lung on CT

CT-based inspiratory and expiratory lung volumes were full-automatically measured by a commercial workstation (NG 1 Ziostation ver. 1.17t; Ziosoft Inc., Tokyo, Japan). Using a three-dimensional technique, the software segmented the lung parenchyma after excluding the chest wall, hilum and central airways.

Maximum lung diameters on CT scans were manually measured on the same workstation by a radiologist (H.T.). The maximum transverse diameter of the lung (inside the chest wall) was measured on an axial CT image that demonstrated the greatest transverse diameter of the lung. Also, the maximum vertical diameter of the lung was defined as the craniocaudal distance from the apex to the bottom of the lung, which was measured by multiplying the number of slices by slice thickness (7 mm). These lung diameters were measured during both inspiration and expiration.

Ultimately, the expiratory/inspiratory (E/I) ratios of lung volume and lung diameters were calculated.

#### (ii) Measurement of the heart on CT

**Fig 1:**
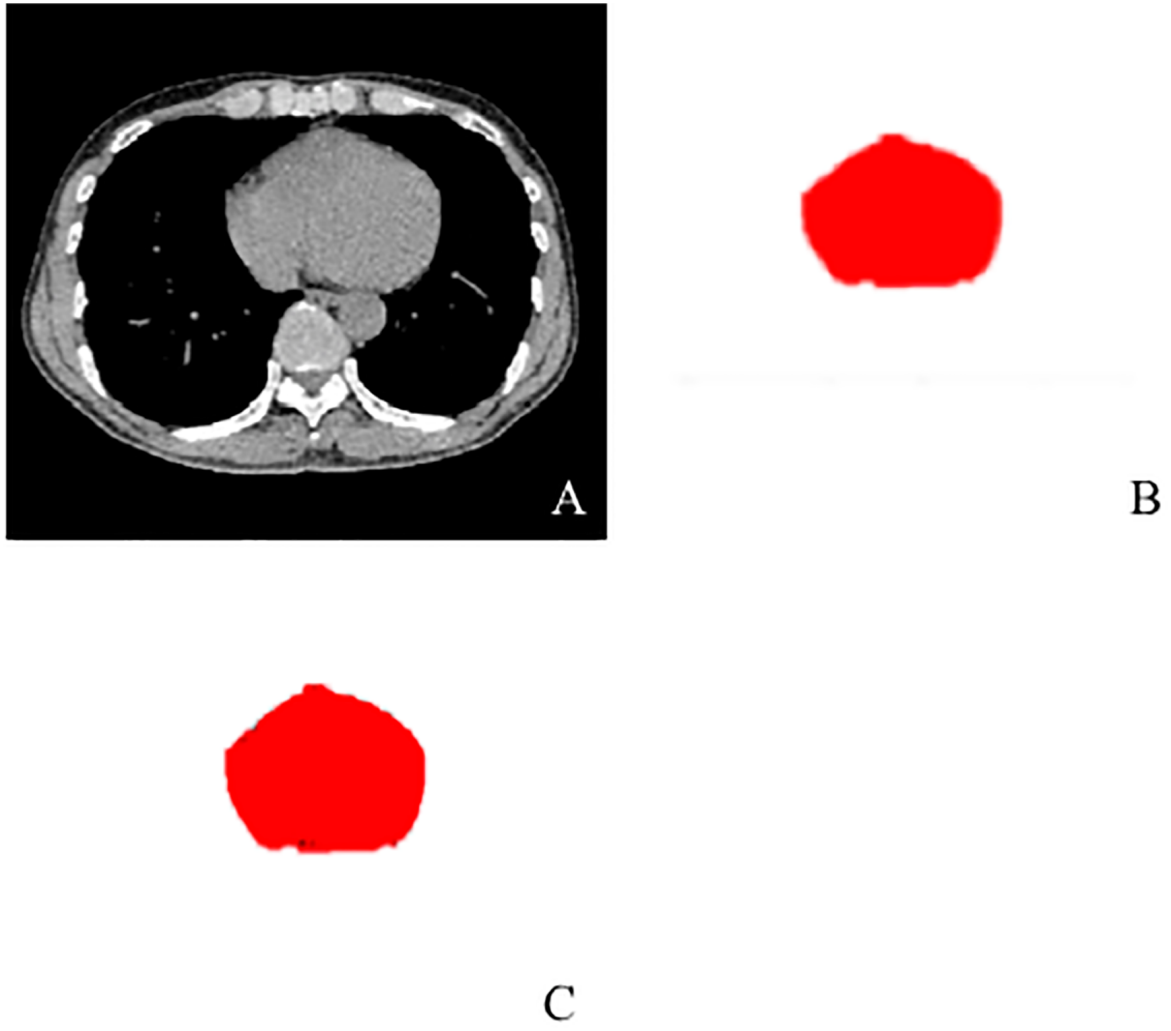
54-year-old male with COPD. The method for measuring cardiac cross-sectional area (CSA) is shown. A CT image with the maximum cardiac CSA was initially selected (**A**). The targeted cardiac area was extracted on the image using a threshold setting (**B**, from 0 to 300 Hounsfield units) to exclude the pericardial fat pad. Black spots included in the cardiac area are fat tissue or artifacts. The cardiac CSA was measured using another threshold setting (**C**, from -100 to 300 Hounsfield units) to include the black spots excluded on the former image.

Cardiac cross-sectional areas (CSA) on CT were semi-automatically measured using open-source software (Image J, version 1.47, Bethesda, MD, USA). In brief, the following process was performed for the measurements (Fig 1): (1) we selected a CT slice that demonstrated the maximum cardiac CSA, which was usually near the diaphragmatic apex; (2) the cardiac boundary was determined by a threshold setting using Hounsfield units (HU) to exclude the pericardial fat pad (from 0 to 300 HU); (3) the targeted cardiac area was extracted using the determined boundary; and (4) cardiac CSA was automatically measured using another threshold setting (from -100 to 300 HU), which included fat tissues or low density artifacts inside the heart. This whole process required approximately three minutes for each CT scan.

For the CTR measurement described below, the maximum transverse cardiac diameter was also measured on the same inspiratory and expiratory CT images, using the same workstation as used for the lung measurements.

Similar to the lung measurements, the E/I ratios of these cardiac dimensions were calculated in order to demonstrate heart size changes between inspiration and expiration.

#### (iii) CTR measurement on CT and chest radiography

CTR was defined as the maximum transverse cardiac diameter divided by the maximum transverse lung diameter. This was calculated on conventional chest radiographs, inspiratory CT scans and expiratory CT scans. CT-based CTR measurements followed the method described in a previous study [12], using the lung and cardiac parameters described above. A set of three CTR measurements was obtained for each patient in this study, including conventional CTR on chest radiography (CXR-CTR), inspiratory CT-based CTR (insp-CT-CTR) and expiratory CT-based CTR (exp-CT-CTR).

### Statistical Analysis

All statistical analyses were performed using JMP 10.0.2 software (SAS Institute, Cary, NC, USA). Data were expressed as the mean ± standard deviation. Comparisons between inspiratory and expiratory measurements were made by the Wilcoxon signed-rank test. Correlations between the E/I ratios of lung and heart measurements and among the three CTR measurements were evaluated by Spearman’s rank correlation analysis.

All *p* values < 0.05 were considered significant.

## Results

### Lung measurements on CT

Table 1 summarizes lung measurements obtained by CT. Lung volume was significantly larger on inspiration (5.03 L ± 1.19) than on expiration (3.65 L ± 0.98) (*p* < 0.0001). The maximum transverse and vertical lung diameters at inspiration were also significantly larger than these at expiration (*p* < 0.0001 for both).

**Table 1:** Lung and cardiac measurements on inspiratory and expiratory CT scans (n = 43).

	Mean ± SD		
	Inspiratory scan	Expiratory scan*	E/I ratio
**Lung measurements**			
Lung volume (L)	5.03 ± 1.19	3.65 ± 0.98	0.73 ± 0.09
Maximum transverse lung diameter (right-left, cm)	26.2 ± 1.7	25.0 ± 1.6	0.95 ± 0.03
Maximum vertical lung diameter (craniocaudal, cm)	34.3 ± 5.3	31.7 ± 4.9	0.90 ± 0.07
**Cardiac measurements**			
Maximum CSA (cm^2^)	7.35 ± 1.57	8.24 ± 7.35	1.13 ± 0.12
Maximum transverse cardiac diameter (right-left, cm)	11.6 ± 1.3	12.1 ± 1.4	1.05 ± 0.06
Cardiothoracic ratio (%)	44.3 ± 5.1	48.8 ± 5.5	—

Definition of abbreviations: CSA = cross-sectional area; E/I = expiratory/inspiratory.

* Significantly different (*p* < 0.0001) from all inspiratory measurements.

### Cardiac measurements on CT

**Fig 2:**
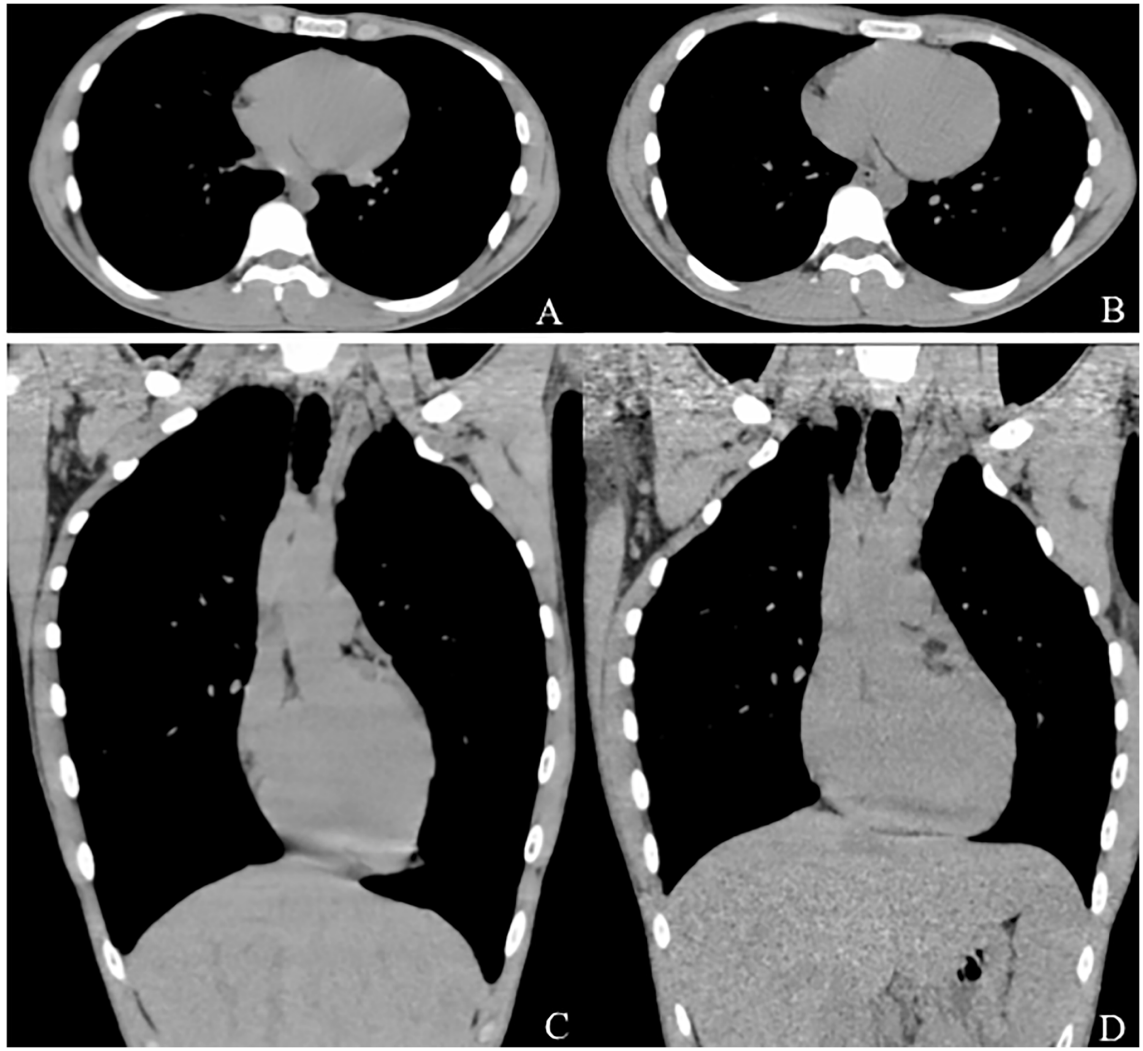
17-year-old male with bronchiectasis. Axial and reconstructed coronal CT images at inspiration (**A** and **C**) and at expiration (**B** and **D**) are shown. The cardiac cross-sectional area and transverse cardiac diameters are smaller at inspiration (**A**) than at expiration (**B**). Based on reconstructed coronal images (**C**, inspiratory; **D**, expiratory), the diaphragm is dislocated downward during inspiration and the cardiac long axis leans vertically (**C**) compared with expiration (**D**). Note that reconstructed coronal images are shown as a reference and were not used for analysis in the study.

The maximum cardiac CSA based on CT was significantly smaller at inspiration (7.35 cm^2^ ± 1.57) than at expiration (8.24 cm^2^ ± 7.35) (*p* < 0.0001, Table 1). Also, the maximum transverse cardiac diameter was smaller at inspiration (11.6 cm ± 1.3) than at expiration (12.1 cm ± 1.4, *p* < 0.0001) (Fig 2).

### Correlations between lung measurements and cardiac measurements

Table 2 demonstrates correlations between lung and cardiac measurements. In brief, the E/I ratios for lung volume and the maximum vertical lung diameter were negatively correlated with the E/I ratios of the cardiac CSA and the transverse cardiac diameter (*p* < 0.01 for all). However, the E/I ratio of the maximum transverse lung diameter was not correlated with the E/I ratio of the cardiac CSA.

**Table 2:** Correlations between the E/I ratios of lung and cardiac measurements.

	Correlation coefficient (ρ)	
	E/I ratio-maximum cardiac CSA	E/I ratio-maximum cardiac transverse diameter
**Lung measurements**		
E/I ratio-lung volume	- 0.529	- 0.595
	(*p* < 0.001)	(*p* < 0.0001)
E/I ratio-maximum transverse lung diameter	0.07	- 0.11
	(NS)	(NS)
E/I ratio-maximum vertical lung diameter	- 0.532	- 0.409
	(*p* < 0.001)	(*p* < 0.01)

Definition of abbreviations: CSA = cross-sectional area; E/I = expiratory/inspiratory; NS = not significant.

### CTR measurements

Table 3 shows the CTR measurements on chest radiography and CT scans. Mean values of exp-CT-CTR, insp-CT-CTR, and CXR-CTR were 48.8% ± 5.5, 44.3% ± 5.1 and 45.3% ± 5.7, respectively. Exp-CT-CTR was significant larger (*p* < 0.0001) than insp-CT-CTR and CXR-CTR (*p* < 0.0001). The difference between insp-CT-CTR and CXR-CTR was also significant (*p* < 0.01). There were strong correlations between each pair of the three CTR measurements (*p* < 0.0001 for all, Table 4).

**Table 3:** Comparisons of the cardiothoracic ratio (CTR) measured on plain chest film, inspiratory chest CT and expiratory chest CT.

	Mean ± SD		
	Plain chest film	Inspiratory CT	Expiratory CT
Cardiothoracic ratio (CTR, %)	45.3 ± 5.7	44.3 ± 5.1*	48.8 ± 5.5**

* Significantly different (*p* < 0.01) from the value on plain chest film.

**Significantly different (*p* < 0.0001) from the values on plain chest film and inspiratory CT.

**Table 4:** Correlations between CTR measurements.

Pairs	Correlation coefficient (ρ)
Insp-CT-CTR and CXR-CTR	0.869
	*(p* < 0.0001)
Insp-CT-CTR and Exp-CT-CTR	0.825
	(*p* < 0.0001)
Exp-CT-CTR and CXR-CTR	0.780
	(*p* < 0.0001)

Definition of abbreviations: CXR = plain chest radiography; insp = inspiratory; exp = expiratory; CTR = cardiothoracic ratio.

## Discussion

In this study, the maximum cardiac CSA and transverse cardiac diameter were larger on expiratory CT than on inspiratory CT, whereas lung volume and lung diameters were larger on inspiratory CT. The changes in lung volume and vertical lung diameter between inspiratory and expiratory CT were negatively correlated with the changes in cardiac CSA and transverse cardiac diameter. Thus, if the patient exhales well, the cardiac CSA and transverse cardiac diameter increase more on expiration. Also, the exp-CT-CTR was larger than the both the insp-CT-CTR and CXR-CTR. These observations suggest that cardiac measurements on chest CT or radiography are significantly influenced by lung volume or the respiratory phase. Furthermore, CTR measurements are affected not only by a change in transverse thoracic diameter, but also by a change in transverse cardiac diameter. To the best of our knowledge, this study is the first to demonstrate a larger cardiac CSA on expiration than on inspiration based on CT. In addition, our new findings include significant correlations between respiratory changes in heart size and lung size, and a difference in CTR between inspiration and expiration.

By using echocardiography and cardiac scintigraphy, it is well known that a decrease in intrathoracic pressure during inspiration causes an increase in venous return and right-sided cardiac volume, which also leads to a decrease in left-sided volume [16–21]. Intravascular and intracardiac pressures and volumes are also important determinants of heart size. However, these previous studies assessed blood volume and sizes of cardiac chambers only; thus, the effect of ventilation on the shape of the heart or transverse cardiac diameter has not been evaluated. Furthermore, if right- and left-sided cardiac volumes change in opposite directions during respiration, the observations in our current study, such as the larger cardiac CSA on expiration, cannot be explained by simple changes in cardiac chamber volumes. Although it is very difficult to explain the larger cardiac CSA or CTR measurements on expiratory based on CT, the following explanation would be plausible: During respiration, muscle contractions or reflections of the diaphragm and external intercostal muscles dramatically change the shapes of the thorax and heart. A previous report showed that the diaphragm is elevated by 6 cm vertically between a full inspiration and expiration, and that vertical movement of the thorax was significantly larger than transverse movement of the thorax [22]. When the diaphragm is dislocated downward during inspiration, the heart becomes narrower and elongated. Furthermore, its left side rotates ventrally and medially, because part of the pericardium attaches to the diaphragm; thus, the cardiac long axis leans in a vertical direction (Fig 2). Conversely, during expiration, the heart becomes wider and shorter; the left side rotates dorsally and laterally. The cardiac long axis leans in a transverse direction due to elevation of the diaphragm [12,22]. Since the superior and inferior vena cava anchor the right atrium, the movement of the right ventricle during respiration is more limited than that of the left ventricle [12]. We believe that based on these physiological observations, the maximum cardiac CSA and diameter were larger on expiration than inspiration, even if there was a decrease in right-sided blood volume.

These changes in transverse cardiac diameters between inspiration and expiration led to inconsistent CTR measurements in this study. In a textbook of pediatric radiology, it was stated that the inclination of the cardiac long axis became < 45 degrees in expiration, resulting in a larger transverse cardiac diameter and CTR measurements [12]. It was also mentioned that CXR-CTR easily reached 0.5 or more by full expiration, which is the general definition of cardiomegaly, even when the inspiratory CXR-CTR was < 0.5. In the current study, we have similarly observed that exp-CT-CTR was significant larger than insp-CT-CTR, and the mean increase in CTR in expiration was approximately 4.5%. Since it has been recommended that an increased CTR should be considered a predictor of increased mortality [1,7–10], such a difference in CTR due to inconsistent breath holding can occur on serial chest radiographs, and it may not be negligible. Therefore, physicians must be aware that CTR can increase if the patient cannot hold their breath properly during scanning, and that lung size should be checked if the CTR suddenly changes on serial images in a single patient, avoiding over- or under-estimation of true heart size.

It is also of interest that CXR-CTR was significant larger than insp-CT-CTR in the current study. Although the difference in CTR between inspiratory CT and radiography was much smaller than that between inspiratory and expiratory CT, or that between radiography and expiratory CT, this phenomenon would suggest that there is a systemic difference between CT and radiography. In a previous study that compared insp-CT-CTR and CXR-CTR, a similar tendency for a CTR difference was observed [11]. Although the reason for a larger CTR based on chest radiography is unclear, this may be partially explained by exclusion of the pericardial fat pad on CT, or differences in body position or x-ray directions between the two modalities. This needs to be investigated in future studies to determine the importance of difference in CTR measured by CT and chest radiography in routine clinical practice.

This study has some limitations. First, since no previous study exists, the optimal density thresholds for measuring the cardiac CSA were not known. Although we attempted multiple settings and determined the thresholds as the best settings, this should be investigated in future studies. Second, since inspiratory and expiratory CT scans were performed to assess various pulmonary or airway diseases in this study, the range of lung volume change and thoracic movement would have been restricted in patients with severe obstructive diseases. In particular, changes in lung volume between inspiratory and expiratory scans would have been very small in some patients with severe COPD due to severe airflow limitation, which should be distinguished from physiological volume changes in patients without airflow limitation. Although we do not believe that it is ethically acceptable, an ideal study population for such physiological research would be healthy controls. Furthermore, since the current study did not target patients with heart disease, we cannot conclude that similar inconsistent CTR measurements truly occur in patients with heart failure. Third, Our study included patients with COPD and did not evaluate the association of the airway obstruction. Since COPD affects lung volume, it would be necessary to correlate airway obstruction with changes of cardiac CSA and CTR between inspiratory and expiratory in future study. Fourth, we did not assess cardiac volumes, since the CT scans were not cardiac or coronary CT using contrast medium and electrocardiogram gating. Thus, it is not clear whether or not enlargement of the cardiac CSA on expiratory CT images correlates with changes in size of the cardiac chambers.

In conclusion, the cardiac CSA and transverse cardiac diameter significantly increase from inspiratory to expiratory CT, and the changes in these cardiac parameters significantly correlate with changes in lung size. The CTR based on CT also significantly changes between inspiration and expiration, which may occur on chest radiographs in patients in the absence of consistent breath-holding and should be distinguished from true improvement or deterioration of cardiomegaly or heart failure.
